# Evaluation of Knowledge, Self-Assessment of Skills and Self-Perception in the Role of Small Animal Practitioner of Veterinary Students Before and After a Structured Clinical Rotation

**DOI:** 10.3390/vetsci13020113

**Published:** 2026-01-24

**Authors:** Katharina Charlotte Jensen, Christin Kleinsorgen, Georga T. Karbe

**Affiliations:** 1 Institute of Veterinary Epidemiology and Biostatistics, School of Veterinary Medicine, Freie Universität Berlin, 14163 Berlin, Germany; 2 Clinic for Cattle, University of Veterinary Medicine Hannover Foundation, 30173 Hannover, Germany; 3 Centre for Teaching, University of Veterinary Medicine Hannover Foundation, 30559 Hannover, Germany; christin.kleinsorgen@tiho-hannover.de; 4 Clinic for Small Animals, University of Veterinary Medicine Hannover Foundation, 30559 Hannover, Germany; georga.karbe@tiho-hannover.de

**Keywords:** veterinary training, teaching, first-day skills, education

## Abstract

Clinical rotations are an integral part of the veterinary curriculum. Their effect on knowledge, skills and self-perception, however, has been poorly investigated. The aim of this study was to evaluate the effect of a structured small animal clinical rotation on veterinary students in these three areas. Participating students were asked to complete an online questionnaire with questions assessing knowledge, skills and self-perception before and after their clinical rotation. A total of 61 students completed the questionnaire before and 43 after the clinical rotation, leading to 41 pre-post matches for self-assessment of skills and self-perception and 39 pairs for knowledge-based questions. The percentage of correctly answered knowledge-based questions increased statistically significantly but only by one correct answer on average. Participants rated their skills in performing specific tasks significantly higher after the clinical rotation compared to before. All participants assessed themselves as competent at history taking, performing a general examination and endotracheal intubation after the clinical rotation. However, 30–40% of participants disagreed at least partly with the sentence that they can perform neurological and ophthalmological examinations as well as interpret blood results on their own after the clinical rotation. Participants rated themselves significantly higher regarding their self-perception in the role of small animal practitioner after the clinical rotation than at the start of the rotation. The study indicated that the clinical rotation improved students’ self-assessment of their skills and attitude but did not lead to a significant improvement in knowledge.

## Simple Summary

As a veterinarian, you need knowledge, skills and also to identify with your role as a veterinarian. Clinical rotations are a part of the veterinary education where students work under the supervision of trained veterinarians with real animals and clients in university clinics. We assessed how a clinical rotation in a small animal clinic influenced the knowledge, the self-confidence concerning certain skills and the self-perception in the role of small animal practitioner. Before the rotation, students correctly answered, on average, 6.8 of 11 questions, and after the rotation they answered 8.0 of 11 questions correctly. The self-perception concerning their skills and their identification with the role of small animal practitioner improved as well. Therefore, this clinical rotation is beneficial for the education of veterinary students. Further studies should assess students’ knowledge, skills and self-perceptions in other settings, as they might differ.

## 1. Introduction

The overall goal of veterinary education is to prepare students for a successful and satisfying career as veterinarians [1]. For this, students will need to feel competent in their abilities by the time they graduate. Competence is a concept that integrates knowledge, skills and attitudes [2]. Knowledge can be taught in various ways and assessed easily by examinations. Concerning the teaching of skills, clinical skill laboratories have been established and more and various simulators have been developed in recent years [3,4]. These lab settings enable students to practice clinical skills incrementally without stressing or harming animals and are suitable for evaluating students’ skills. Nevertheless, “real life” experiences with real animals and clients are needed to prepare students for their careers [5]. Teaching and assessing the third dimension of competence—attitude—is considerably more challenging. Attitude is part of the professional identity—the set of values and priorities that are meaningful to the individual as a veterinarian [6,7]. Allister [7] stated that without the ability to fit in and work with the norms and values held by others in the profession, veterinary graduates leave university ill-equipped for their professional life. Even though there are approaches to incorporate the teaching of veterinary professionalism in veterinary education [8], there is still room for improvement regarding defining the skills and attributes of the veterinary professional and the methods for how to teach these [9]. Concepts to teach “professional identity” or “attitudes” are challenging as students need to experience the profession and reflect on their experiences ideally in small group sessions [9].

Moreover, the self-efficacy (here also referred to as self-perception) in a (new) role can be seen as a part of attitude. It has been shown that the way someone thinks of his or her abilities to learn or perform actions influences learning, motivation, as well as future behavior, e.g., goal persistence or task avoidance (Bandura’s theory of self-efficacy [10,11]). Self-perception integrates different sources: performance (e.g., past success/failures, need for assistance), observational comparison (e.g., to other students), social feedback (e.g., by teachers or supervisors) and physiological states (feelings when doing something) [11]. Self-efficacy contributes to medical students’ preparedness for clinical practice [12] and is linked to higher levels of resilience in veterinary medicine [13].

Intra- and extra-mural clinical trainings are fundamental components of the veterinary curriculum as students can familiarize themselves with professional life, apply the knowledge they have acquired and learn and improve first-day skills [2,14]. However, clinical rotations are difficult in terms of learning success as they cannot be standardized.

In Germany, the veterinary education includes a so-called practical year (5th year). Besides extra-mural elective practical training and training in food hygiene and veterinary public health, students have to complete an intra-mural clinical rotation with a duration of 10–14 weeks (460 h). In Hannover, students can choose to complete their clinical rotation in the following veterinary teaching hospitals: (a) cattle, (b) small ruminants and pigs, (c) small animals, (d) horses or (e) small mammals, reptiles and birds.

Up until now, evaluations of these intra-mural clinical rotations have mainly been conducted in order to assess the students’ satisfaction and to identify the need for improvement. These evaluations have not been published. The effect of the clinical rotation on the students has not yet been assessed. The aim of this study was to evaluate a structured intra-mural clinical rotation of 460 h in small animal medicine in terms of the knowledge, skills and self-perception of students in the role of small animal practitioner. Therefore, changes in correctly answered questions (knowledge), in subjective self-confidence in performing ten different tasks (skills) and in agreement with ten sentences assessing five subscales of self-perception (identification with the role as small animal practitioner) were measured. Moreover, as secondary aims, the questionnaires were analyzed in order to assess their internal consistency as well as to determine the correlations among the three concepts.

## 2. Materials and Methods

### 2.1. Ethics

Participation was voluntary; no personal information was gathered, so answers could not be assorted to individual persons. The official data protection officer W. Rottwinkel (wolfgang.rottwinkel@tiho-hannover.de) of the Stiftung Tierärztliche Hochschule Hannover approved this study, considering it to be in accordance with data privacy standards, i.e., ethical guidelines regarding research data of human participants. Since the data protection officer is not tied to an ethical review board, no official approval code was granted. As the checklist provided by the central Ethics Committee did not suggest any ethical concerns, ethical approval was not necessary.

Participants were informed that the aim of this study was to evaluate the effect of the clinical rotation in terms of their knowledge, skills and self-perception in the role of small animal practitioner. Hence, informed consent to participate in the study was obtained from all survey participants and data were processed and evaluated anonymously.

### 2.2. Description of the Clinical Rotation

The clinical rotation consisted of a minimum of 460 h over an 11–12-week period. Twenty students (in one case, twenty-one students) were enrolled per clinical rotation.

Students rotate in pairs and each pair starts this cycle on a different week (Table 1). All students participate in an initial clinical-skills-lab week where they revise performing skills such as performing a general physical exam and specialty exams (orthopedic, neurologic, dermatologic and ophthalmologic) as well as other tasks such as history taking, placing catheters, endotracheal intubation, etc. In addition, the students have feedback sessions at the end of the first week, at the halfway point and at the end of the 12th week (except those on night shift at that time). The student-to-veterinarian ratio ranges from 2:3 to 2:5 per service.

Students engaged in hands-on training with multiple patients daily. Students were treated as junior colleagues and had full access to the patient record system. Students were involved with client communication, medical history, clinical examination, clinical reasoning, application of evidence-based medicine and critical thinking, developing a diagnostic and treatment plan, treatment and documentation.

Cases and completed tasks were recorded by the students in a logbook with a minimum requirement of completed tasks. Clinicians signed off on completed tasks. Logbooks were reviewed at the end of the rotation or in between if needed. Logbooks automatically showed progress, based on the minimum required practical activities in the respective clinic. In addition, student performance was assessed weekly by the supervising staff. Based on these evaluations and the OSCE, students received structured and individual feedback, verbally and written, at least twice during their clinical rotations.

As a more detailed example, students on clinical rotation in the anesthesia and intensive care services became involved in the daily management of elective and emergency procedures. During didactic rounds, the cases of the day and their possible problems and special considerations were discussed with the students. Students were assigned to 1 to 3 cases per day and took part in the case management, i.e., they were involved from premedication and anesthesia induction to maintenance and monitoring, the recovery phase and planning of postoperative pain management and possibly intensive care measures, imaging procedures, surgery techniques, etc. On assigned cases, students practiced physical examinations, blood sampling, placement of catheters, endotracheal intubation, local and regional anesthesia techniques, handling of an anesthesia device and monitoring equipment, set-up of infusion sets, handling of infusion devices and writing patient records. Depending on the case load, students were also involved in euthanasia cases and the preceding decision-making.

### 2.3. Development of Questionnaire

The questionnaire was developed based on a literature review and consensus among investigators. For knowledge, senior clinicians of different disciplines who regularly supervise students on their clinical rotation were asked to provide questions that are suitable for assessing the knowledge of students in their 5th year of studies. Skills were chosen based on previous German research projects [15,16] within the university, as well as own experiences and the feedback of colleagues. For self-perception, we found no specific validated instruments that measure self-perception or self-efficacy in the role of veterinarian. General scales measuring in self-efficacy as a personality state appeared not suitable as we aimed to assess it in this specific context. Therefore, we adapted a scale exploring the self-perception of pupils in intermediate grades as readers [11]. This scale is focused on persons in a specific educational context and their affections while being in the new role. We stuck, therefore, to the concept of self-perception instead of the more general term self-efficacy. In this scale, self-perception consists of five subscales: *General* and *Specific Progress* (perception of past performance compared to present performance)*, Observational Comparison* (comparison to other students), *Social Feedback* (feedback from supervisors) and *Physiological State* (internal feelings when being in the role).

A total of 31 questions were devised and then assigned to one of three main question groups:

(1) Questions testing knowledge: 11 questions in single-best answer format, with one correct, three false and the option “I don’t know” (Table 1). Question and answer formats are similar to those in the “progress test in veterinary medicine” [17]. They were not derived from that pool.

(2) Questions for self-assessment of practical skills: 10 rating questions on a Likert-type scale from 1 = “I do not agree at all” to 5 = “I totally agree”.

(3) Questions regarding self-perception: 10 items adapted from the Reader Self-Perception Scale (SPS, [14]) with two questions each for the dimensions *General Progress*, *Specific Progress*, *Comparison through Observation*, S*ocial Feedback* and *Physiological State*. The assignment of items to the subscales, as well as examples from the original scale, can be found at the end of the questionnaire in the Supplementary Material S1. Each item was to be answered on the same Likert-type scale as the questions referring to skills.

Questions and answers were implemented into LimeSurvey© (LimeSurvey Community Edition Version 6.13.2+250506) and pilot tested by ten colleagues working in different fields of veterinary medicine for quality assurance. Minor changes were made after pilot testing (wording). After the first group of students, analyses revealed that three knowledge-based questions were answered correctly by almost all the students. Therefore, answer options were changed by trying to add stronger distractors. The questions were not changed to ensure comparability.

### 2.4. Data Collection

Students were asked on their first day of the clinical rotation (start of the skills lab week) to fill out the questionnaire with their own mobile devices or with the device of the instructor. Each student received a small card with a QR access code to the questionnaire and a unique animal species (name and photo). The animals were used to keep students anonymous while allowing before and after clinical rotation comparisons per individual. Students were asked to keep the card or to take a photo of it. On the last day of the clinical rotation, students were asked again to fill out the questionnaire using the same animal identification.

Data collection took place between April 2024 and February 2025.

### 2.5. Analyses

Data is provided as Supplementary Material (S2). Data was exported in Microsoft Excel (version 2408) and imported to SAS Enterprise Guide (version 7.1) for data management and statistical analyses. Some of the analyses (e.g., calculation of Kelley’s Discrimination Index) were conducted using IBM SPSS (version 29.0.0.0). Figures were created using Microsoft Excel.

If participants did not answer one question, the value was imputed by the mode of this question. This means that we filled in the answer chosen by most of the participants for this item if an answer was missing. If we had discarded these persons, we would have lost not only one timepoint but also the subsequent timepoint and this would diminish statistical power. Instead of losing 10 pairs, we imputated 11 data points (two missing items from one person; 0.3%). Imputation was performed before the calculation of sum scores. Four students did not answer the last page assessing self-perception, so the number of answers varies.

Sums of the questions regarding self-assessment of skills and self-perception were calculated. Moreover, the percentage of correctly answered knowledge questions was determined per student. For knowledge questions, the participants also had the option to choose “I don’t know”. For calculating the percentage of correctly answered questions, these were regarded as not correctly answered. Kelley’s Discrimination Index (KDI) was calculated for knowledge items. This old, simple and robust index is defined as the difference between the best 27% of participants and the poorest 27% of participants [18]. A common interpretation of the KDI is as follows: <0 unacceptable, 0–0.2 poor, 0.21–0.29 acceptable, 0.30–0.39 good and >0.40 (up to max. 1) excellent.

For self-perception in the role of small animal practitioner, Cronbach’s Alpha was used to assess internal consistency. A value higher than 0.7 could be interpreted as acceptable, even though the number of items also influences the value [19]. The self-perception scale had two items in every dimension. To test if this assumption also applies to these data, we built a correlation matrix using Spearman’s correlation coefficient. For the self-assessment of skills as well as the knowledge part, Cronbach’s Alpha or other measures of internal consistency were not assessed, as we did not assume these different skills were one-dimensional.

For the knowledge items, we calculated the item difficulty (percentage of participants who answered this item correctly).

Descriptive analyses were carried out, mainly displaying numbers and percentages. To test whether students assessed themselves better after the clinical rotation, paired tests were calculated. Therefore, a smaller dataset was used as only those participants who answered the questionnaire twice and whose questionnaires could be matched could be interpreted. Here, only persons who participated in the survey before and after the clinical rotation were included to rule out any bias caused by persons who did not finish the study. Difference scores were checked for normal distribution using the Kolmogorov–Smirnov test. In case of normal distribution, a paired t-test was used, and in case of non-normally distributed differences, a Wilcoxon test for paired samples was used. Moreover, means with confidence intervals were calculated for matched pairs. *p*-values < 0.05 were considered statistically significant.

Finally, we also investigated the associations among knowledge, skills and self-perception. We calculated Spearman’s correlation coefficient for the sum of correctly answered knowledge questions, the sum of agreement with skills and the sum of agreement with the self-perception items. These calculations were based on the large data set including all responses. *p*-values have not been corrected for multiple testing.

## 3. Results

### 3.1. Participants

All students, in total 61, answered the questionnaire at the beginning of their clinical rotation (group A: *n* = 20, group B: *n* = 20, group C: *n* = 21). On the last day of their clinical rotation, 43 students filled out the questionnaire (group A: *n* = 16, group B: *n* = 15, group C: *n* = 12). All students completed the entirety of the clinical rotation. Due to the rotational schedule, including night-time duties, not all students were present on the final day to complete the questionnaire. A few students could not complete the questionnaire because they had forgotten or misplaced their animal identification cards. Since animals were handed out randomly without record keeping, identification could not be retrieved. Others did not fill out the questionnaire as participation was voluntary. Four students did not answer the questions regarding knowledge or forgot/neglected to submit the last page. So, the total number of students is 100 for the knowledge part. For knowledge, 39 matches were analyzed and 41 matches were analyzed for self-assessment of skills and self-perception.

### 3.2. Knowledge

**Figure 1:**
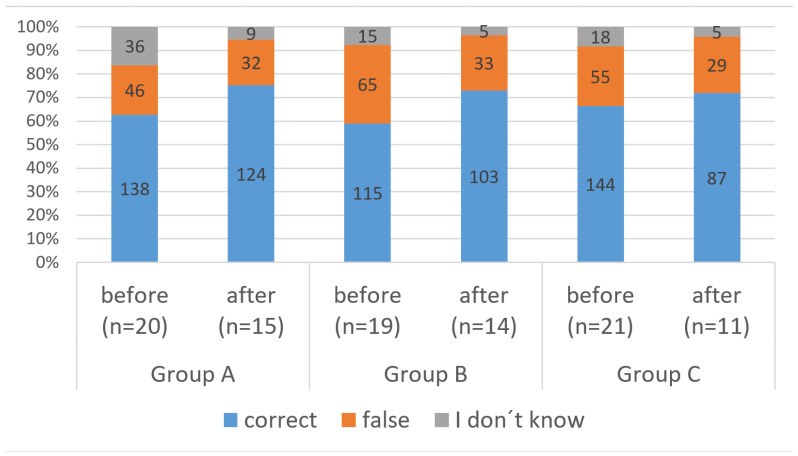
Percentage of correct and false answers to questions assessing the knowledge of veterinary students before and after a clinical rotation in small animal medicine.

The questions, item difficulties and KDI are displayed in Table 2. Six items discriminated excellently among the best and poorest participants, three items discriminated well and two items discriminated badly. No item had a negative KDI. Before their clinical rotation, students answered 6.75 questions (*n* = 60) correctly, and after their clinical rotation, they answered 7.95 questions (*n* = 40) correctly. This finding was consistent among the three groups (Figure 1). A paired *t*-test confirmed a statistically significant but small increase in correctly answered questions (t(38) = 4.1, *p* < 0.001; |*d*| = 0.66). The mean difference for matched pairs (*n* = 39) was 1.13 (95%-CI: 0.58–1.68). This increase was more attributable to a decrease in “I do not know” answers than to a decrease in incorrect answers (Figure 1). This might be attributable to a testing effect.

### 3.3. Self-Evaluation of Skills

**Figure 2:**
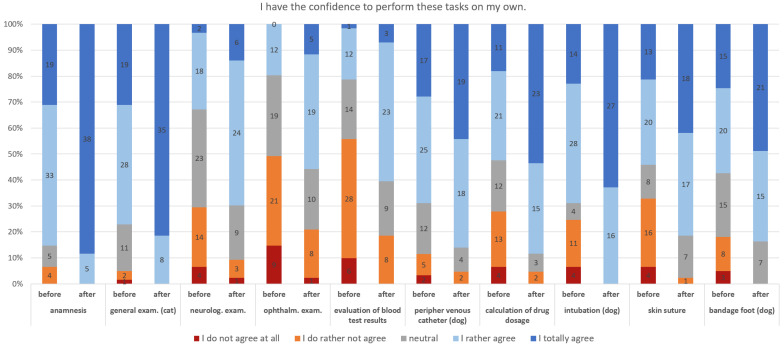
Self-assessment of skills of veterinary students before and after a clinical rotation in small animal medicine.

Regarding the sum of overall skills, students assessed themselves significantly better after the clinical rotation compared to the initial assessment (t(40) = 8.46; *p* < 0.001). This effect can be regarded as large (|*d*| = 1.32; [20]). The mean of the difference between the sum of scores before and after the rotation was 7.00 (95%-CI: 5.33–8.67, *n* = 43). Regarding single skills (Figure 2), differences are visible between tasks. Neurological and ophthalmological examinations, as well as the interpretation of blood test results, seemed to be the most challenging skills, as students rated themselves worst at this skill (before and after the clinical rotation). On the other hand, anamnesis, general examination and intubation seemed to be less challenging. Here, all students agreed that they had the confidence to perform these tasks after the internship (Figure 2).

### 3.4. Self-Perception in the Role of Small Animal Practitioner

Internal consistency of this part of the questionnaire was high (Cronbach’s Alpha: 0.86). The correlation of items is displayed in Table 3. Items of the same dimension were expected to correlate more strongly than with items of other dimensions. This was only the case concerning *Occupational Comparison* (OC). For instance, the item GP1 (“I feel competent as a small animal practitioner”) was more highly correlated with item PS1 (“I feel safe as a small animal practitioner.”) than with item GP2 (“I think, I will be a competent small animal practitioner.”). Interestingly, the item PS2 (“I think, examining and treating cats and dogs is great”) showed the lowest correlation with all other items.

**Figure 3:**
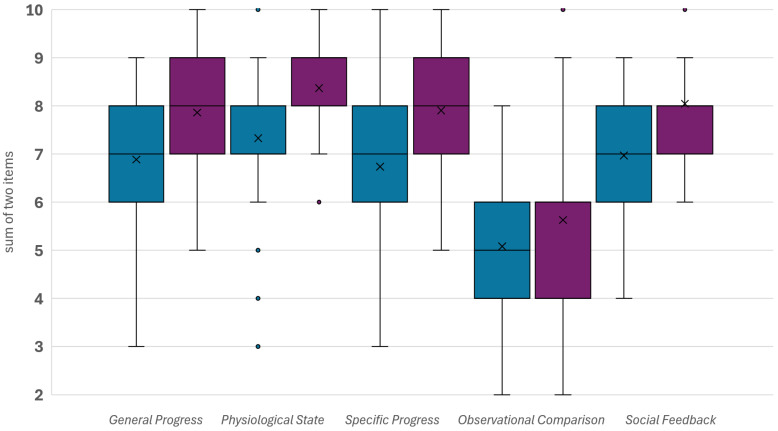
Self-perception of veterinary students before (blue; *n* = 61) and after (purple; *n* = 43) a clinical rotation in small animal medicine. Higher scores indicate a higher level of agreement/confidence. Means are marked with X.

Students showed, in general, high agreement regarding the dimensions *General* and *Specific Progress* as well as *Physiological State* and *Social Feedback* (Figure 3). Concerning *Observational Comparison*—the comparison of students’ own knowledge and skills with other students’ knowledge and skills—lower levels of agreement were observed, and students rated themselves as average.

Regarding the sum of dimensions, a statistically significant increase (mean = 4.7) was observed after the clinical rotation compared to before (Wilcoxon test for paired samples: *p* < 0.001). This effect can be regarded as large (*r* = 0.84). The median of the difference of the sums before and after the clinical rotation was 4.00 (25% quartile: 2; 75%-quartile: 7; *n* = 43).

### 3.5. Correlation Among the Three Dimensions

The sum of correctly answered questions had a correlation coefficient of 0.31 (95%-confidence interval [95%-CI]: 0.12–0.48) with the sum of skill items and a coefficient of 0.27 (95%-CI: 0.07–0.45) with the sum of self-perception items; the sum of skill items and the sum of self-perception items showed a stronger correlation (0.72 [95%-CI: 0.61–0.80]).

## 4. Discussion

This is one of the first studies evaluating a clinical rotation for veterinary students in Germany. A small but significant improvement was observed in students’ knowledge, their self-assessment of clinical skills and their professional self-perception as small-animal practitioners.

### 4.1. Limitations

This evaluation study included an assessment before and after the clinical rotation, allowing tracking of the development of participants. A control group would have been beneficial to estimate whether the repeated questioning influenced the answers of participants. Causal comparisons require treated and control groups to be balanced on pre-treatment covariates; without such baseline equivalence, estimated effects are confounded by pre-existing differences [21]. Such a control group was not feasible as other groups of students who chose clinical rotation, e.g., in large animal medicine, would have differed substantially from our cohorts in regard to knowledge, skills and self-perception.

The high rate of dropouts is surely a limitation of this study. To rule out the bias caused by dropouts, we conducted paired tests concerning the sums of correctly answered questions and self-assessment of skills, as well as the total score of self-perception in the role of small animal practitioner. We conducted further sensitivity analyses to analyze if the persons who dropped out differed from those who took part a second time. Therefore, we compared the sum of correctly answered questions, the sum of the self-assessment of skills and the sum of the questions of self-perceptions before the clinical rotation using a Mann–Whitney U-test. Participants who dropped out later, did not differ concerning knowledge (*p* = 0.372) and self-perception (*p* = 0.553), but assessed themselves worse concerning clinical skills before the clinical rotation (*p* = 0.014; mean rank of persons who dropped out: 22.7; mean rank of persons who also participated after the rotation: 34.7). This difference might be attributable to a lower level of competence or different personality traits, like self-esteem. As there was no difference concerning the self-perception in the role of small animal practitioner, the latter hypothesis seems unlikely.

Another limitation is that the quality of the questionnaire was improvable: the questions regarding knowledge were too easy to answer at the beginning, as participants answered nearly 7 out of 11 questions correctly before the clinical rotation. Therefore, we modified three questions by changing the distractors in these questions. We assume that this influenced the results only slightly, as the percentage of correctly answered questions did not differ significantly among groups (Figure 1). The KDI indicated a good or excellent discriminatory power of 9 of 11 items.

Concerning the questionnaire measuring self-perception, correlation analyses did not display the intended subscales. However, the ten questions of this part of the questionnaire showed high internal consistency, so the scale as a whole might be useful to assess students’ self-perception in the role of small animal practitioner. However, further studies examining self-perception should carefully consider using this scale or should at least investigate construct validity.

Two of three concepts were assessed based on self-reports. These were also strongly correlated, indicating that other factors—like personality traits—might influence both. For the concept of self-perception in the role of small animal practitioner, other assessment methods are hardly available. For the assessment of skills, an observed measure of competence would have been more valid as self-rated assessments often differ from assessments made by trained persons [22]. Even though self-confidence differs from objective assessments, self-confidence helps students to feel prepared. Self-confidence in the ability to perform these tasks is therefore a key part of the transition from student to clinical practice [23].

Results are not transferable to other contexts since clinical rotations, as well as the previous education and attitudes of students, differ from university to university. We pursued three cohorts of veterinary students. As these cohorts showed similar results, we conclude that the observed effects are consistent within this setting.

### 4.2. Development of Knowledge

In this study, the development of knowledge, skills (assessed as self-confidence in performing certain tasks) and self-perception in the role of small animal practitioner was assessed. This is in contrast to most published studies evaluating clinical rotations or internships in veterinary medicine, as these typically focus on expectations and satisfaction with the program [24,25,26].

The increase in knowledge was statistically significant, but on average, students answered only one more question correctly after the clinical rotation compared to before. Even though some researchers would consider the observed effect as large [20], the interpretation should also consider whether the dependent variable is easy or difficult to influence [27]. As students had to answer the same questions before and after the rotation, an increase in correct answers was to be expected. It is also likely that some students learned the right answer by talking about it in the group or looking it up. Another reason for the slight increase might also be the already high rate of correct answers before the rotation (mean: 7 of 11). Students also had to have passed exams in internal medicine, surgery and reproduction before their practical year, so they were probably well-prepared regarding knowledge. Future studies should include more difficult or more practice-related questions when assessing knowledge. Interestingly, the increase was not due to a reduction in wrong answers but to a smaller proportion of participants who chose the option “I do not know”. After the clinical rotation, students may have felt more pressure to answer correctly and guessed instead of admitting their uncertainty. This finding is in line with another study assessing the learning progress of veterinary students in biochemistry [28], in which the increase in right answers was also mainly attributable to a decrease in “I don’t know” answers and was interpreted as an increase in confidence [28].

### 4.3. Self-Evaluation of Skills

In the last two decades, research concerning the use of simulators for teaching clinical skills has increased [5]. These simulators may provide advantages—such as repeatability and animal welfare [29]. Nevertheless, when students reach a certain level of experience, they need to gain experience in “real-life” scenarios with living animals and clients for the best learning outcomes [5]. Clinical rotations provide these scenarios. However, repeatability and standardization of learning experiences are limited as not all students can witness the same situations and procedures [30]. Therefore, the evaluation of learning experiences in clinical settings is very important [31]. In this study, students had the opportunity to practice some of the skills, e.g., intubation, using simulators and then perform them on living animals (or with real clients). This is an ideal procedure to ensure the best educational value for students, while minimizing the risk involved for pets [30]. Indeed, our results indicate a significant increase in self-confidence when performing different tasks. The differences concerning the difficulty of the tasks are in line with another recently published German study referring to external rotations [16]. There, students reported feeling less confident in performing an ophthalmological examination, a bit more confident in performing a neurological examination and quite confident in general clinical examinations [16]. Like in that study, all or most students in this study felt confident concerning anamnesis, intubation, suturing and setting a peripheral venous catheter. These tasks are important as they are frequently performed as first-day competencies and are also required to perform first aid [2].

### 4.4. Self-Perception in the Role as Small Animal Practitioner

The self-perception in the role of small animal practitioner has been assessed with a scale based on a questionnaire for pupils or adults learning to read [11,32]. There are questionnaires available that have already been used to measure self-perception in veterinary medicine, even after clinical rotations [33]. However, those questionnaires focus more on practical skills and general abilities, while we focused on self-perception in the role of veterinarian and thus used a scale measuring self-perception. Therefore, we designed a new questionnaire based on a validated instrument, the SPS [11]. Even though the intended five subscales could not be confirmed, the whole scale showed high internal consistency. In future studies, this questionnaire should be further developed and validated in order to have an instrument to assess self-perception in the role of veterinarian.

A small but significant increase in self-efficacy was assessed, indicating that students became more familiar and confident in their role. This is important as the transition from studying to practicing can be challenging [34]. Younger veterinarians are at a higher risk of being affected by distress [35,36], while self-efficacy is positively associated with resilience [13]. In the study by Allister [37], the identity of being a veterinarian was identified as a central factor in the transition from student to practice. Even though most participants stated that they wanted to become a vet from an early age, they did not identify with this role as they were not independent and still needed support [37]. In the study by Schull et al. [33], comparable results were observed concerning the preparedness and self-confidence of students regarding their role as veterinarians. Confidence is not only an internal state but is also the most important attribute from the point of view of clients [38].

### 4.5. Correlation Among the Three Dimensions

Regarding the correlations among knowledge, skills and self-perception, it became evident that the two concepts that were assessed based on a self-assessment were highly correlated. Knowledge—assessed more objectively based on the percentage of correctly answered questions—correlated less strongly with the two self-assessed scores. This might be caused either by a common method bias [39] and/or by a global positive (or negative) view of themselves.

## 5. Conclusions

Following a clinical rotation in their final year, a significant increase was noticeable in veterinary students’ self-perception of skills, as well as in their self-perception in the role of small animal practitioner, compared to before the clinical rotation. The increase in knowledge was also statistically significant but not relevant educationally and could have arisen from a testing effect. This study is one little step toward evaluating clinical rotations and how they relate to learning success. Most of the research conducted prior to this study has focused on the perception of clinical rotations or on clinical skills labs. Therefore, concepts and tools to evaluate internships or clinical rotations in regard to learning success are still lacking. It is important not to focus only on knowledge and skills but also to assess the change in self-confidence, professionalism and identification with the profession in order to facilitate the transition from veterinary student to veterinarian. Moreover, we recommend including other data sources, like diaries, interviews or OSCE scores, in future studies to assess skills as well as self-perception.

## Figures and Tables

**Table 1:** Schedule of veterinary students during their clinical rotation in the small animal clinic. All students start their rotation in the clinical skills lab and then start in different sections in pairs.

Weeks	Monday	Tuesday	Wednesday	Thursday	Friday	Saturday	Sunday
1	Clinical Skills Lab	Clinical Skills Lab	Clinical Skills Lab	Clinical Skills Lab	OSCE Exam		
2	Reproductive Medicine	Reproductive Medicine	Reproductive Medicine	Reproductive Medicine	Reproductive Medicine		
3	Anesthesia	Anesthesia	Anesthesia	Anesthesia	Anesthesia		
4	Internal Medicine	Internal Medicine	Internal Medicine	Internal Medicine	Internal Medicine	Wards day	Wards day
5	Neurology	Neurology	Physical therapy	Neurology	Neurology		
6	Dermatology	Dermatology	Dermatology	Emergency	Dermatology		
7	Radiology	Radiology	Orthopedics	Orthopedics	Orthopedics		
8	Soft Tissue	Soft Tissue	Soft Tissue	Soft Tissue	Emergency		
9	Ophthalmology	Ophthalmology	Oncology	Oncology	Oncology	Wards night 5 p.m.–2 a.m.	Wards night 5 p.m.–2 a.m.
10	off	Dentistry	Elective	Dentistry	Elective		
11	Wards Medicine	Wards Medicine	Lab	Wards Medicine	Wards Medicine		
12	Nights 5 p.m.–2 a.m.	Nights 7 p.m.–4 a.m.	Nights 5 p.m.–2 a.m.	Nights 7 p.m.–4 a.m.	Nights 5 p.m.–2 a.m.		

**Table 2:** Knowledge test: overview of questions, item difficulties and discrimination indices.

Question	% of Correct Answers (Total)	Before Clinical Rotation	After Clinical Rotation	Kelley’s Discrimination Index
A German Shepherd is presented to the emergency service in lateral recumbency—what is my first step?	67	72	60	0.19
I am monitoring the general anesthesia of a 2-year-old cat and notice that the blood pressure is steadily dropping. What should I do first?	65	65	65	0.50
Bella, a seven-year-old female, spayed Maltese, presents with bilateral mucoid ocular discharge. What is the first diagnostic step after a general examination?	76	63	95	0.56
Max, a Labrador, is lame at the right hind limb, his stifle joint feels thickened and is painful on hyperextension. What is my working diagnosis?	64	62	68	0.56
Susie, a 10-year-old mixed-breed dog, has a subcutaneous mass. How do I proceed?	73	67	83	0.38
A bright eight-week-old puppy is presented for an initial examination. On auscultation of the heart you hear a left-sided, continuous grade V/VI heart murmur. What is the suspected diagnosis?	65	60	73	0.38
Garfield the cat is reported to be drinking and urinating large amounts. Which of these laboratory values should I pay particular attention to?	60	52	73	0.06
I have removed an intestinal foreign body from cat Cookie via an enterotomy. How soon after surgery can we offer Cookie food?	23	12	40	0.13
I would like to carry out a neurological examination on a dog. Unfortunately, the dog is aggressive. Which part of the examination is most important?	57	60	53	0.69
A cat is presented to the emergency service with open mouth breathing. What do I do first?	87	87	88	0.31
For a dog with paralysis, the 5-finger rule can be used to identify the most likely differential diagnoses. What are the components of this rule?	86	77	100	0.44

**Table 3:** Correlations of items measuring self-perception in the role of small animal practitioner of veterinary students before and after a clinical rotation in small animal medicine (*n* = 104).

GP1	GP1									
GP2	0.53 ***	GP2								
PS1	0.75 ***	0.40 ***	PS1							
PS2	0.22 *	0.29 *	0.22 *	PS2						
SP1	0.70 ***	0.54 ***	0.70 ***	0.17	SP1					
SP2	0.44 ***	0.26 **	0.43 ***	0.30 **	0.38 ***	SP2				
OC1	0.49 ***	0.40 ***	0.46 ***	0.08	0.46 ***	0.34 **	OC1			
OC2	0.42 ***	0.40 ***	0.46 ***	0.20*	0.49 ***	0.32 **	0.77 ***	OC2		
SF1	0.40 ***	0.28 **	0.41 ***	0.18	0.40 ***	0.36 **	0.56 ***	0.42 ***	SF1	
SF2	0.38 ***	0.39 ***	0.29 **	0.24 *	0.33 **	0.30 **	0.25 ***	0.10	0.23 *	SF2

Spearman’s correlation coefficient. * *p* < 0.05; ** *p* < 0.01; *** *p* < 0.001. GP1/2 = items on general progress, PS1/2 = items on *Physiological State*, SP1/2 = items on *Specific Progress*, OC1/2 = items on *Observational Comparison*, SF1/2 = items on *Social Feedback*. Items of the same dimension (highlighted in green) were expected to show the highest correlation coefficients.

## Data Availability

The original contributions presented in this study are included in the article and Supplementary Material S2. Further inquiries can be directed to the corresponding author.

## Supplementary Materials

The following supporting information can be downloaded at: https://www.mdpi.com/article/10.3390/vetsci13020113/s1, S1. questionnaire, S2 data.

## Abbreviations

The following abbreviations are used in this manuscript:

KDI	Kelley’s Discrimination Index
PY	Practical year
SPS	Self-Perception-Scale
