# Supplementary material for: Evaluation of Knowledge, Self-Assessment of Skills and Self-Perception in the Role of Small Animal Practitioner of Veterinary Students Before and After a Structured Clinical Rotation
Source: Vet Sci. 2026 Jan 24;13(2):113. doi: 10.3390/vetsci13020113 (PMC12945202; doi:10.3390/vetsci13020113)
Supplement: Supplementary file 1 [file vetsci-13-00113-s001.zip › Questionnaire_evaluation-20260114.pdf]

Dear students,

Welcome to the Small Animal Clinic! You can look forward to some intensive and informative weeks ahead.

With the help of this survey, we would like to examine how your knowledge, skills, and self-perception have changed in your role as a small animal veterinarian. Please answer the questions based on your gut feeling—there is often no right or wrong answer. Participation is, of course, voluntary.

Kind regards,

Charlotte, Christin and Georga

Part A: Identification

What animal did you draw?

- Beluga whale
- Alpine newt
- Bluethroat
- Blue-black carpenter bee
- Brown long-eared bat
- Cafeteria single-celled organism
- Bottlenose seahorse
- Moose
- Yellow-eyed penguin
- House sparrow
- House spider
- Grey seal
- Royal albatross
- Copper-striped pipefish
- Mississippi alligator
- Moselle Apollo butterfly
- Nine-banded armadillo
- Barn owl
- Bull beetle
- Squirrel monkey
- Raccoon
- Lesser flamingo
- Sand lizard

Part B: Self-Perception

Please indicate to what extent you agree with the following statements.

	1 – I do not agree at all	2 – I do rather not agree	3 – neutral	4 – I rather agree	5 – I totally agree
I think I will become a competent small animal veterinarian.					
I like examining and treating dogs and cats.					
I have the necessary knowledge and skills to examine and treat small animals.					
I have more veterinary knowledge than my fellow students.					
I feel confident in my role as a small animal veterinarian.					
My fellow students think I will be a good small animal veterinarian.					
In my role as a small animal veterinarian, I feel competent.					
I have more veterinary skills than my fellow students.					
I can lead conversations with pet owners in an empathetic and structured manner.					
The veterinarians who mentor me give me the feeling that I will be a good veterinarian.					

Part C: Veterinary Skills

Please indicate to what extent you agree with the following statements.

I am confident in my ability to independently …	1 – I do not agree at all	2 – I do rather not agree	3 – neutral	4 – I rather agree	5 – I totally agree
… take a patient history.					
… perform a general physical examination on a cat.					
… perform a neurological examination on a pet.					
… perform an ophthalmological examination on a pet.					
… evaluate blood test results.					
… place a peripheral venous catheter in a dog.					
… calculate the correct dose required for an infusion or medication.					
… intubate a dog.					
… suture skin.					
… bandage a paw correctly on dogs.					

Part D: Knowledge-Check

Please answer the questions spontaneously!

There is always one correct answer.

(correct answers are highlighted in green)

1) A German Shepherd is presented to the emergency service in lateral recumbency - what is my first step?
   - Test pupil reflexes
   - Auscultate the heart
   - Assess mucous membranes
   - Measure body temperature
   - I don´t know

2) I am monitoring the general anesthesia of a 2-year-old cat and notice that the blood pressure is steadily dropping. What should I do first?
   - Check depth of anesthesia
   - Auscultate heart
   - Administer glycopyrrolate
   - Increase inhalation anesthesia
   - I don´t know

3) Bella, a 7-year-old female, spayed Maltese, presents with bilateral mucoid ocular discharge. What is the first diagnostic step after a general examination?*
   - Slit lamp examination
   - Performing the Schirmer tear test
   - Measuring intraocular pressure
   - Ophthalmoscopic examination
   - I don´t know

4) Max, a Labrador, is lame in the right hind limb, his stifle joint feels thickened and is painful on hyperextension. What is my working diagnosis?
   - Ruptured patellar tendon
   - Ruptured lateral collateral ligament
   - Ruptured lateral meniscus
   - Ruptured cranial cruciate ligament
   - I don´t know

* for this questions, distractors were partly different during the first round.

5) Susie, a 10-year-old mixed-breed dog, has a subcutaneous mass. How do I proceed?*
   - Fine needle aspiration
   - Prescribe antibiotics
   - Ultrasound examination of the mass
   - Surgical removal
   - I don´t know

6) A bright, 8-week-old puppy is presented for an initial examination. On auscultation of the heart you hear a left-sided, continuous grade V/VI heart murmur. What is the suspected diagnosis?
   - Pulmonary artery stenosis
   - Mitral valve insufficiency
   - Atrial septal defect
   - Persistent ductus arteriosus
   - I don´t know

7) Garfield, a cat, is reported to be drinking and urinating large amounts. Which of these laboratory values should I pay particular attention to?*
   - Calcium
   - Hematocrit
   - Lipase
   - Leukocytes
   - I don´t know

8) I have removed an intestinal foreign body from cat Cookie via an enterotomy. How soon after surgery can we offer Cookie food?
   - As soon as she can get up
   - 24 hours post-op
   - Up to 48 hours post-op only water
   - Small meals after 36 hours
   - I don´t know

9) I would like to carry out a neurological examination on a dog. Unfortunately, the dog is aggressive. Which part of the examination is most important?
   - Proprioception
   - Spinal reflexes
   - Cranial nerve function
   - Adspection
   - I don´t know

* for this questions, distractors were partly different during the first round.

10) A cat is presented to the emergency service with open mouth breathing. What do I do first?
   - Quick blood sampling for blood gas analysis
   - Prompt orthogonal view thoracic radiograph
   - Place directly in the oxygen cage
   - Sedation and endotracheal intubation
   - I don´t know

11) For a dog with paralysis, the 5-finger rule can be used to identify the most likely differential diagnoses. What are the components of this rule?
   - Chronicity, progression, painfulness, symmetry of clinical signs, neuroanatomical localization
   - Signalment, feeding history, general examination, neurological examination, laboratory findings
   - Airway, breathing, circulation, neurological status (disability), exposure/environment
   - Ability to walk, proprioception, deep pain, spinal reflexes, cutaneous Trunci cut-off
   - I don´t know

Thank you very much!

* for this questions, distractors were partly different during the first round.

Table 1: Self-perception items in this study, the assignment to subscales and examples from the Reader Self-Perception Scale [1,2] that inspired our items.

**Subscale**	**Items of the self-perception as a small animal veterinarian**	**Examples of the Reader Self-Perception Scale [1,2]**
Physiological state	I like examining and treating dogs and cats.<br>I feel confident in my role as a small animal veterinarian.	I like to read out loud.<br>I feel good inside when I read.
Occupational comparison	I have more veterinary knowledge than my fellow students.<br>I have more veterinary skills than my fellow students.	I seem to know more words than other kids when I read.<br>When I read, I can figure out words better than other kids.
Social Feedback	My fellow students think I will be a good small animal veterinarian.<br>The veterinarians who mentor me give me the feeling that I will be a good veterinarian.	People in my family think I am a good reader.<br>My teachers think that my reading is fine.
General Progress	I think I will become a competent small animal veterinarian.<br>In my role as a small animal veterinarian, I feel competent.	I read better now than I could before.<br><br>I am getting better at reading.
Specific Progress	I have the necessary knowledge and skills to examine and treat small animals.<br>I can lead conversations with pet owners in an empathetic and structured manner.	I can figure out words better than I could before.<br>I understand what I read better than I could before.

1. Henk, W. A.; Melnick, S. A. The Reader Self-Perception Scale (RSPS): A new tool for measuring how children feel about themselves as readers. *The Reading Teacher* **1995,** *48* (6), 470–482.
2. Henk, W. A.; Marinak, B. A.; Melnick, S. A. Measuring the reader self-perceptions of adolescents: Introducing the RSPS 2. *Journal of Adolescent & Adult Literacy* **2012,** *56* (4), 311–320. DOI: 10.1002/JAAL.00144.

* for this questions, distractors were partly different during the first round.
